# Dkk1 Stabilizes Wnt Co-Receptor LRP6: Implication for Wnt Ligand-Induced LRP6 Down-Regulation

**DOI:** 10.1371/journal.pone.0011014

**Published:** 2010-06-08

**Authors:** Yonghe Li, Wenyan Lu, Taj D. King, Chia-Chen Liu, Gautam N. Bijur, Guojun Bu

**Affiliations:** 1 Department of Biochemistry and Molecular Biology, Drug Discovery Division, Southern Research Institute, Birmingham, Alabama, United States of America; 2 Departments of Pediatrics, St. Louis Children's Hospital, Washington University School of Medicine, St. Louis, Missouri, United States of America; 3 Department of Cell Biology and Physiology, Washington University School of Medicine, St. Louis, Missouri, United States of America; 4 Department of Psychiatry and Behavioral Neurobiology, University of Alabama at Birmingham, Birmingham, Alabama, United States of America; New York State Institute for Basic Research, United States of America

## Abstract

**Background:**

The low density lipoprotein receptor-related protein-6 (LRP6) is an essential co-receptor for canonical Wnt signaling. Dickkopf 1 (Dkk1), a major secreted Wnt signaling antagonist, binds to LRP6 with high affinity and prevents the Frizzled-Wnt-LRP6 complex formation in response to Wnts. Previous studies have demonstrated that Dkk1 promotes LRP6 internalization and degradation when it forms a ternary complex with the cell surface receptor Kremen.

**Methodology/Principal Findings:**

In the present study, we found that transfected Dkk1 induces LRP6 accumulation while inhibiting Wnt/LRP6 signaling. Treatment with Dkk1-conditioned medium or recombinant Dkk1 protein stabilized LRP6 with a prolonged half-life and induces LRP6 accumulation both at the cell surface and in endosomes. We also demonstrated that Kremen2 co-expression abrogated the effect of Dkk1 on LRP6 accumulation, indicating that the effect of Kremen2 is dominant over Dkk1 regulation of LRP6. Furthermore, we found that Wnt3A treatment induces LRP6 down-regulation, an effect paralleled with a Wnt/LRP6 signaling decay, and that Dkk1 treatment blocked Wnt3A-induced LRP6 down-regulation. Finally, we found that LRP6 turnover was blocked by an inhibitor of caveolae-mediated endocytosis.

**Conclusions/Significance:**

Our results reveal a novel role for Dkk1 in preventing Wnt ligand-induced LRP6 down-regulation and contribute significantly to our understanding of Dkk1 function in Wnt/LRP6 signaling.

## Introduction

The canonical Wnt signaling pathway is involved in various differentiation events during embryonic development, and when aberrantly activated it can lead to tumor formation. Central to the Wnt signaling pathway is the stabilization of cytosolic β-catenin, which binds transcription factors of the T-cell factor/lymphoid enhancing factor (TCF/LEF) family leading to the transcription of Wnt target genes [1]–[4]. In the absence of Wnt ligands, β-catenin is phosphorylated by a multi-protein complex that marks it for ubiquitination and degradation by the proteasome. This β-catenin degradation complex contains the adenomatous polyposis coli (APC) tumor suppressor, the scaffold protein Axin, glycogen synthase kinase 3β (GSK3β), and casein kinase 1 (Ck1). The action of this complex is inhibited upon Wnt binding to its receptors. The low density lipoprotein receptor-related protein 5 (LRP5) and LRP6 are essential co-receptors for Wnt signaling [1]–[4]. By binding to the seven-transmembrane-domain receptor frizzled (Fz), and LRP5/LRP6, Wnt ligands stabilize cytoplasmic β-catenin [1]–[4].

LRP5 and LRP6 are closely related cell surface receptors that belong to the expanding low density lipoprotein receptor (LDLR) family [1], [5], and are subjected to modulation by secreted antagonists [1], [5]. Distinct from several families of secreted Wnt antagonists that bind Wnts, including the secreted Fz-related protein (sFRP) family and Wnt inhibitory factor 1 (Wif1), Dkk1 does not bind Wnt but is a high affinity ligand for LRP5/6 [6]–[8]. Dkk1 inhibits Wnt signaling by preventing the Fz-Wnt-LRP5/6 complex formation in response to Wnt [8]. In *Xenopus* and in mammals, the Dkk family includes Dkk1, Dkk2, Dkk3 and Dkk4, which exhibit distinct expression patterns and properties [9], [10]. In addition to LRP5/6, Dkk1 binds to Kremen1 and Kremen2, two related single-transmembrane-domain proteins [11]–[13]. Since Dkk1 can stimulate LRP6 internalization upon Kremen2 overexpression, it was proposed that by binding to both LRP6 and Kremen2, Dkk1 induces LRP6 internalization from the cell surface, thereby attenuating Wnt signaling [12].

Ligand-induced receptor down-regulation plays a key role in regulating the propagation and duration of growth factor receptor signaling, thereby avoiding aberrant cellular stimulation [14], [15]. In the present studies, we characterized the roles of Dkk1 in LRP6-mediated Wnt signaling. Our results reveal a novel function for Dkk1 in Wnt ligand-induced LRP6 down-regulation and Wnt/LRP6 signaling.

## Results

### Opposing effects of Dkk1 on LRP6 protein level and Wnt/LRP6 signaling

**Figure 1:**
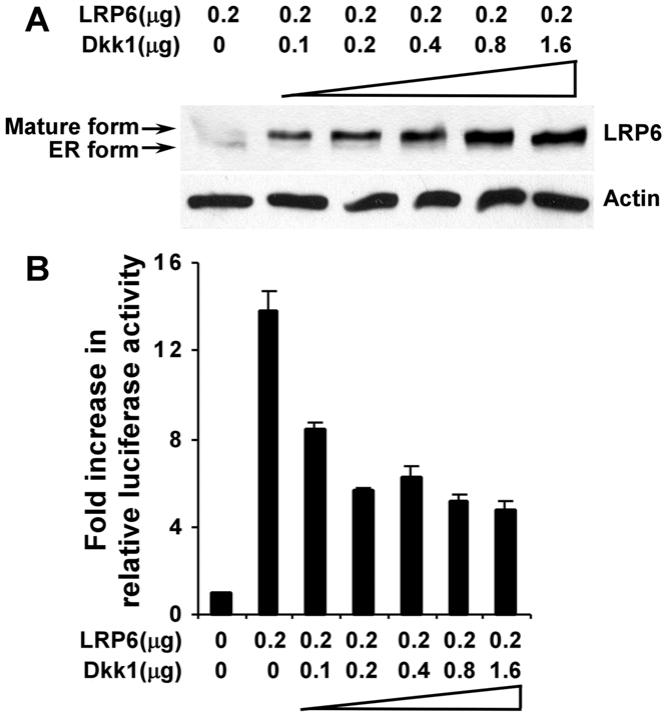
Opposing effects of Dkk1 on LRP6 protein level and Wnt/LRP6 signaling. (A) HEK293 cells were co-transfected with indicated amounts of Dkk1 and Myc-tagged human LRP6 plasmids. Total plasmid levels were balanced with the empty vector. After 48 h, the level of LRP6 was examined by Western blotting using the anti-Myc antibody. (B) HEK293 cells were co-transfected with indicated amounts of LRP6 and Dkk1 plasmids together with 0.1 µg of the TOP-FLASH TCF luciferase construct and 0.1 µg of β-galactosidase-expressing vector. After 48 h, the luciferase activity was determined with normalization to the activity of the β-galactosidase.

Previous studies have shown that Dkk1 binds to the Wnt co-receptor LRP6 and prevents the formation of active Fz-Wnt-LRP6 receptor complexes, thus blocking the canonical Wnt pathway [8]. To investigate how binding of Dkk1 to LRP6 influences receptor trafficking and turnover, we performed co-transfection of Myc-tagged LRP6 cDNA with human Dkk1 into HEK293 cells. The levels of LRP6 were analyzed 48 h after transfection by Western blotting using Myc antibody, which revealed two bands of LRP6 (Figure 1A). The lower band represents the ER precursor form that lacks complex sugar modifications, while the upper band represents the mature form of the receptor [16]. As seen in Figure 1A, Dkk1 co-expression significantly increased the steady state level of mature LRP6. The level of LRP6 protein was gradually enhanced with increasing amounts of Dkk1 cDNA being transfected (Figure 1A).

Since it was surprising to find that Dkk1 increased the steady state level of LRP6, we performed TOP-FLASH TCF transactivation assays to confirm that Dkk1 inhibits the LRP6-mediated Wnt signaling under these conditions. As seen in Figure 1B, expression of LRP6 cDNA alone increased TCF activity about 14-fold when compared to vector-transfected cells. Inhibition of LRP6-mediated Wnt signaling was observed when Dkk1 was co-expressed with LRP6, with maximal inhibition observed with equivalent amounts of cDNA for Dkk1 and LRP6.

### Dkk1 acts on cell surface to induce LRP6 accumulation

**Figure 2:**
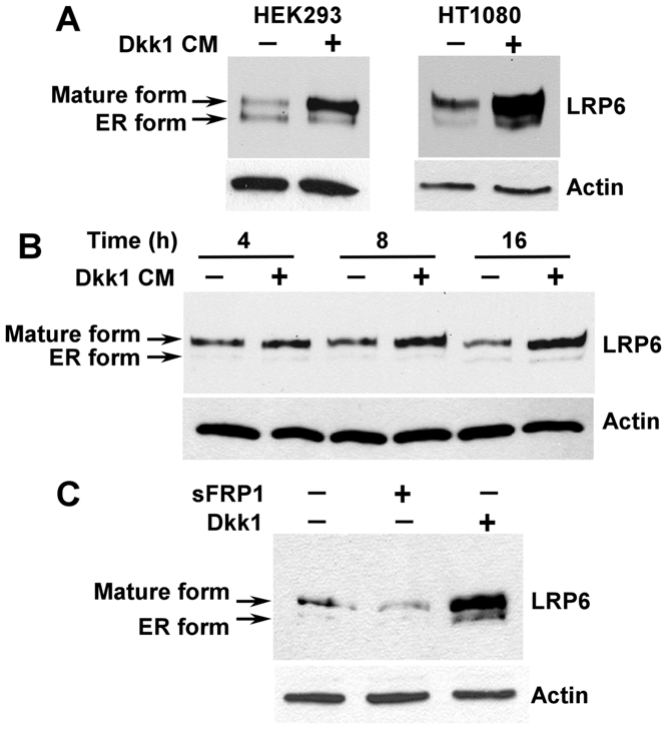
Exogenously added Dkk1 is sufficient to enhance LRP6 protein level. (A) HEK293 cells in 6 well plates were transiently transfected with 0.8 µg of Myc-tagged human LRP6 plasmid. Twenty four hours following transfection, cells were cultured with Dkk1 CM (+) or vector-transfected control CM (−) for additional 24 h. The level of LRP6 was then examined by Western blotting using the anti-Myc antibody. Similarly, HT1080 cells stably transduced with HA-tagged LRP6 in 6-well plates were cultured with Dkk1 CM or control CM for 24 h, and the level of LRP6 was then examined by Western blotting with the anti-HA antibody. (B) HT1080 cells stably transduced with HA-tagged LRP6 were cultured with Dkk1 CM or control CM for 4, 8, or 16 h. The level of LRP6 was then examined by Western blotting with the anti-HA antibody. (C) HT1080 cells stably transduced with HA-tagged LRP6 in 6 well plates were cultured with recombinant Dkk1 protein (1 µg/ml) or recombinant sFRP1 (1 µg/ml) for 24 h. The level of LRP6 was then examined by Western blotting with the anti-HA antibody. Samples were also probed with anti-actin antibody to verify equal loading.

To test whether Dkk1 acts on the cell surface LRP6, as opposed to those in the secretory pathway, we examined the effect of exogenously added Dkk1 on LRP6 protein level. HEK293 cells transiently transfected with Myc-tagged LRP6 were incubated for 24 h with conditioned media (CM) from either vector-transfected or Dkk1 cDNA-transfected cells, and the levels of LRP6 were examined by Western blotting. As seen in Figure 2A, Dkk1 CM, but not control vector CM, enhanced LRP6 protein level. Similar results were obtained when HT1080 cells stably transduced with LRP6 were tested (Figure 2A). To analyze the kinetics of this process, we performed a similar experiment with HT1080-LRP6 cells by incubating them with Dkk1 CM for 4, 8, or 16 h. As seen in Figure 2B, an increase of LRP6 is cumulative over time.

To eliminate potential effects of other proteins in the Dkk1 CM on LRP6 expression, and to study the role of another type of Wnt signaling antagonists on LRP6 protein level, we examined the effects of human recombinant Dkk1protein and human recombinant sFRP1 protein on LRP6 stabilization. As seen in Figure 2C, Dkk1, but not sFRP1, enhanced LRP6 protein level. All together, these results suggest that Dkk1 applied to the cell surface can achieve the same effects on LRP6 expression as Dkk1 cDNA co-transfection.

### Dkk1 stabilizes LRP6

**Figure 3:**
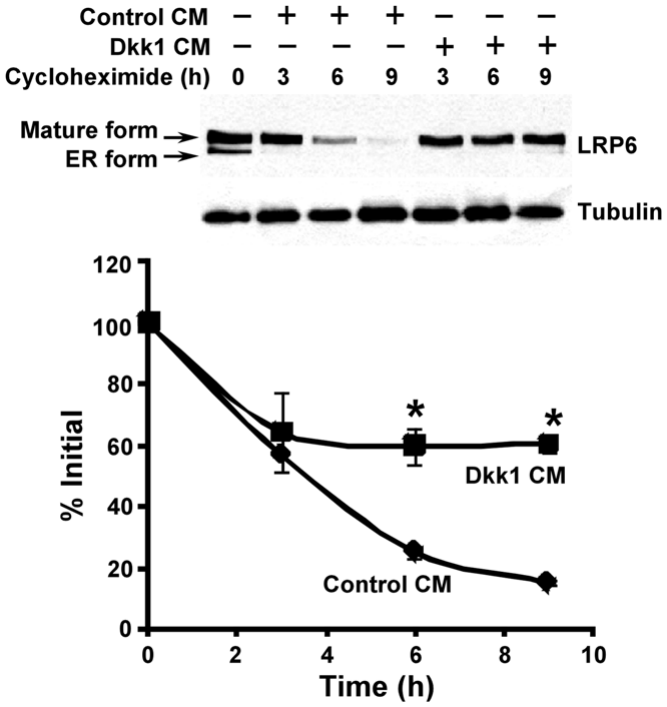
Dkk1 stabilizes LRP6. HT1080 cells stably transfected with HA-tagged LRP6 were incubated with 5 µg/ml of cycloheximide in the presence of 25% of Dkk1 CM or 25% of control CM for 0, 3, 6 or 9 h. Cells were then harvested, and the level of LRP6 was examined by Western blotting. Samples were also probed with anti-tubulin antibody to verify equal loading. The pixels for each band were measured, normalized and plotted. Data are mean values of three independent experiments with the SE values indicated by error bars. *P<0.05 versus corresponding control value.

To dissect the molecular mechanism underlying the effect of Dkk1 on LRP6 protein level, we studied LRP6 turnover. HT1080 cells stably transduced with HA-tagged LRP6 were treated with Dkk1 CM in the presence of cycloheximide, a protein synthesis inhibitor. As seen in Figure 3, in the absence of Dkk1 treatment, LRP6 was rapidly degraded with a half-life of less than 4 h. However, treatment with Dkk1 significantly stabilized LRP6. There was still about 60% LRP6 remaining after the cells were treated with Dkk1 for 9 h (Figure 3). This prolongation in L RP6 half-life suggests that Dkk inhibits LRP6 turnover.

### Kremen2 induces LRP6 down-regulation and Wnt/LRP6 signaling inhibition

**Figure 4:**
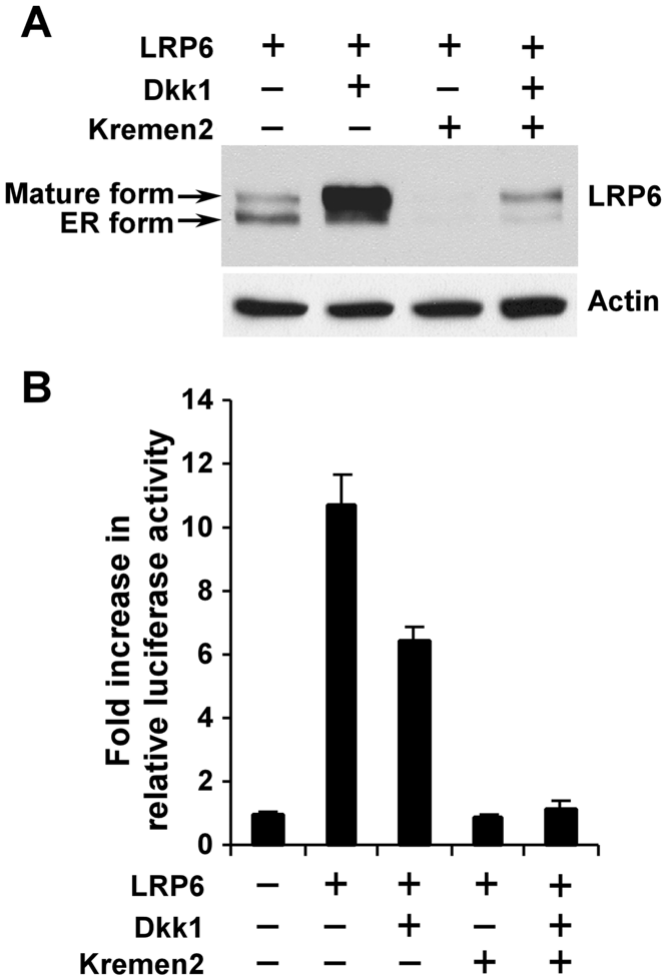
Kremen2 induces LRP6 turnover and Wnt/LRP6 signaling down-regulation. (A) HEK293 cells were transiently co-transfected with 0.2 µg of Myc-tagged human LRP6 plasmid, and 0.8 µg of human Dkk1, 0.8 µg of mouse Kremen2, or both Dkk1 and Kremen2 plasmids. After 48 h post-transfection, the level of LRP6 was examined by Western blotting with the anti-Myc antibody. Samples were also probed with the anti-actin antibody to verify equal loading. (B) HEK293 cells were transiently co-transfected with 0.2 µg of Myc-tagged human LRP6 plasmid, 0.8 µg of human Dkk1, 0.8 µg of mouse Kremen2, or both Dkk1 and Kremen2 plasmids together with 0.1 µg of the TOP-FLASH TCF luciferase construct, and 0.1 µg of β-galactosidase-expressing vector. After 48 h, the luciferase activity was determined with normalization to the activity of the β-galactosidase.

It has been shown that Kremen2 forms a ternary complex with Dkk1 and LRP6 and induces rapid endocytosis and removal of LRP6 from the cell surface [11]–[13]. Since our studies above have demonstrated a role for Dkk1 in stabilizing LRP6, we were interested in understanding the relationship between Dkk1 and Kremen on the regulation of LRP6. We performed co-transfection experiments of LRP6 cDNA in the absence or presence of cDNAs for Dkk1, Kremen 2, or both, followed with analysis of steady state levels of LRP6, as well as LRP6-mediated Wnt signaling. Similar to the results above, co-transfection of Dkk1 alone stabilized LRP6 (Figure 4A), but decreased Wnt/LRP6 signaling (Figure 4B). Interestingly, co-transfection of Kremen 2 alone is sufficient to abolish LRP6 expression (Figure 4A) and Wnt/LRP6 signaling (Figure 4B). Co-transfection of Dkk1 and Kremen 2 gave similar results as Kremen 2 alone, suggesting that the effect of Kremen 2 on LRP6 stabilization and Wnt/LRP6 signaling is dominant over that of Dkk1.

### Wnt3A induces LRP6 down-regulation

**Figure 5:**
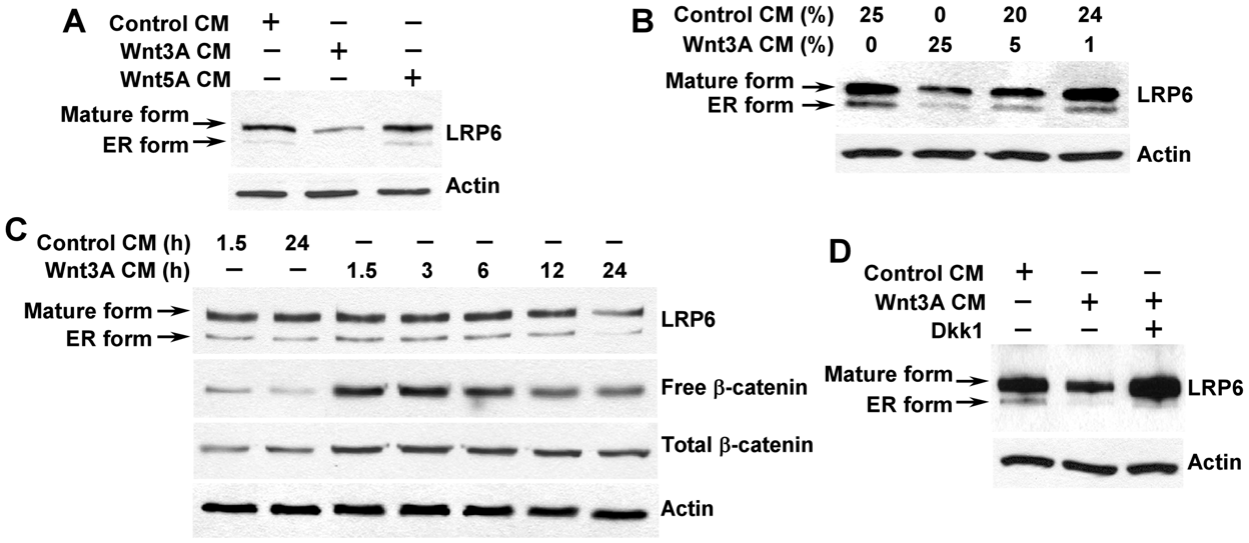
Dkk1 blocks Wnt3A-induced LRP6 down-regulation. (A) HT1080 cells stably transduced with HA-tagged LRP6 in 6 well plates were incubated with 25% of Wnt3A CM, Wnt5A CM or L cell control CM for 24 h. The level of LRP6 was then examined by Western blotting with anti-HA antibody. (B) HT1080 cells stably transduced with HA-tagged LRP6 in 6 well plates were incubated with 0–25% of Wnt3A CM for 24 h. The level of LRP6 was then examined by Western blotting. (C) HT1080 cells stably transfected with HA-tagged LRP6 in 6 well plates were incubated with 25% of Wnt3A CM for 1.5–24 h. The levels of total cellular LRP6, cytosolic free β-catenin and total cellular β-catenin were examined by Western blotting. (D) HT1080 cells stably transduced with HA-tagged LRP6 in 6 well plates were incubated with 25% of L cell control CM, 25% of Wnt3A CM, or 25% of Wnt3A CM with 1 µg/ml of recombinant Dkk1 protein for 24 h. The level of LRP6 was then examined by Western blotting with the anti-HA antibody. Samples were also probed with the anti-actin antibody to verify equal loading.

Ligand-induced receptor degradation has emerged as an important cell biological process that down-regulates the activity of various signaling pathways [14], [15]. We hypothesized that Wnt binding to LRP6 also induces receptor endocytosis and subsequent degradation. Previous studies have demonstrated that Wnt3A binds to LRP6 [17], while Wnt5A, a noncanonical Wnt ligand, binds to frizzled receptors but not LRP6 [18]–[20]. Thus, we examined the effects of Wnt3A and Wnt5A on cellular levels of LRP6. Our results showed that Wnt3A CM, but not Wnt5A CM, reduced LRP6 levels after 24 h treatment (Figure 5A), and that Wnt3A-induced LRP6 down-regulation was dose dependent (Figure 5B). We then studied the connection between LRP6 down-regulation and Wnt/LRP6 signaling decay. Uncomplexed cytosolic β-catenin (free β-catenin) is an active form of β-catenin which can enter the cell nucleus and associate with the transcription factors of the TCF/LEF family, leading to the transcription of Wnt target genes. We examined free β-catenin levels to monitor Wnt/LRP6 signaling after Wnt3A stimulation. It was found that Wnt3A-induced LRP6 down-regulation was time dependent and was roughly parallel to the decrease of free β-catenin (Figure 5C). The level of LRP6 protein started to decrease after 12 h of Wnt3A CM treatment, while the level of free β-catenin started to decrease after 6 h of Wnt3A CM treatment (Figure 5C). All together, these results suggest that LRP6 down-regulation and Wnt/LRP6 signaling decay occur after activation of Wnt/LRP6 signaling when the cells were treated with Wnt3A.

### Dkk1 blocks Wnt3A-induced LRP6 down-regulation

By binding to the Wnt co-receptor LRP6, Dkk1 prevents Wnt binding to LRP6 [8]. Having established that there is a Wnt3A-induced LRP6 down-regulation and Wnt/LRP6 signaling decay after Wnt3A treatment, we then investigated the effects of Dkk1 on Wnt3A-induced LRP6 down-regulation. As expected, we found that co-incubation of recombinant Dkk1 protein with Wnt3A CM blocks Wnt3A-induced LRP6 down-regulation (Figure 5D).

### Dkk1 induces LRP6 accumulation, and blocks Wnt3A-induced LRP6 down-regulation on the cell surface and in endosomes

**Figure 6:**
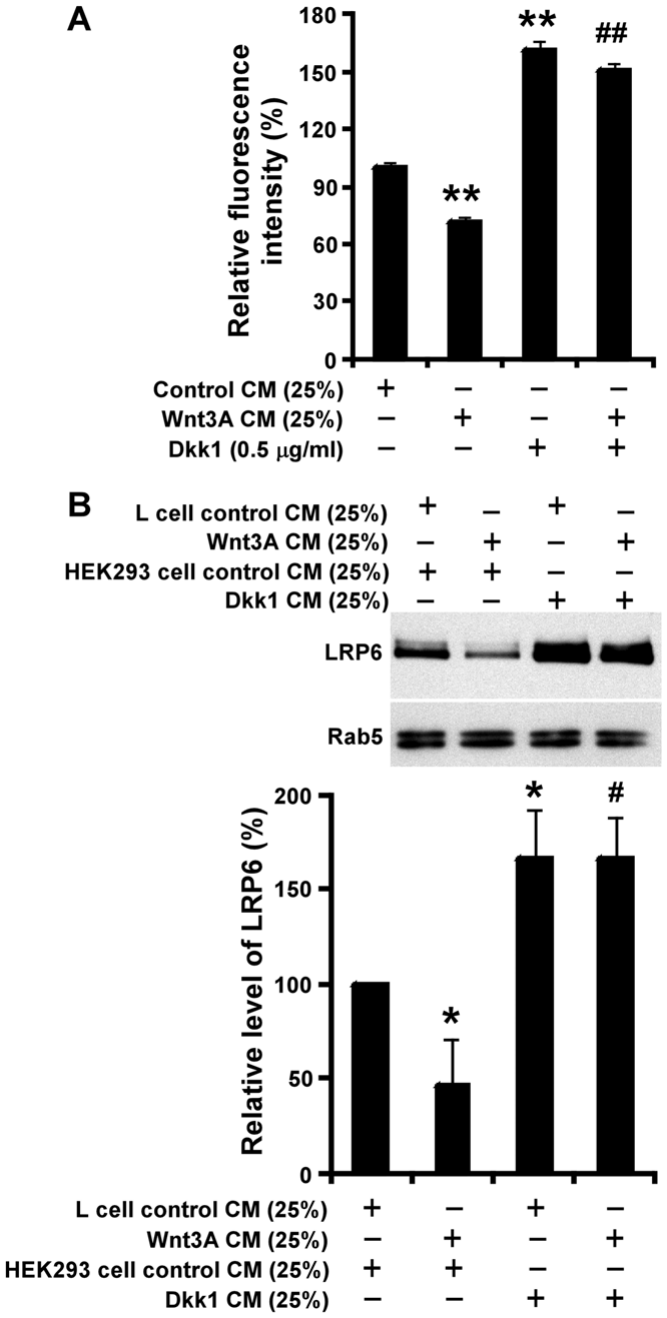
LRP6 levels on cell surface and in endosomes after Dkk1 and Wnt3A treatment. (A) HT1080 cells stably transduced with HA-tagged LRP6 were incubated with 25% of L cell control CM, 25% of Wnt3A CM, 0.5 µg/ml of recombinant Dkk1 protein, or 25% of Wnt3A CM plus 0.5 µg/ml of recombinant Dkk1 protein for 24 h. Cell surface LRP6 was labeled with the anti-HA antibody and detected with goat anti-mouse IgG-FITC by FACS. Each value represents the difference between total and background fluorescence intensities and is the average of triple determinations with the SE indicated by error bar. **P<0.01 compared to L cell control CM-treated cells; ^##^P<0.01 compared to L cell control CM-treated cells and Wnt3A CM-treated cells. (B) HT1080 cells stably transduced with HA-tagged LRP6 were incubated with control CMs, 25% of Wnt3A CM, 25% of Dkk1 CM, or Wnt3A CM plus Dkk1 CM for 24 h. Levels of LRP6 in endosomes were examined by Western blotting with the anti-HA antibody. Samples were also probed with the anti-Rab5 antibody to verify equal loading and endosome preparation. The pixels for each band were measured, normalized and plotted. Data are mean values of four independent experiments with the SE values indicated by error bars. *P<0.05 compared to L cell control CM-treated cells; ^#^P<0.05 compared to L cell control CM-treated cells and Wnt3A CM plus Dkk1C CM-treated cells.

LRP6 cDNA stably transduced in HT1080 cells has an HA-tag at its N-terminus [21]. We next compared cell surface LRP6 levels via FACS analysis with anti-HA antibody to test whether there is a change of LRP6 levels on the cell surface after Dkk1 and/or Wnt3A treatment. Non-specific staining of the anti-HA antibody was undetectable in untransduced HT1080 control cells [21]. Treatment of HT1080 LRP6 cells with Dkk1 recombinant protein (0.5 µg/ml) for 24 h resulted in a 62% increase, while Wnt3A treatment resulted in a 28% decrease, of cell surface LRP6 levels. Furthermore, co-incubation of recombinant Dkk1 protein with Wnt3A CM blocked Wnt3A-induced LRP6 down-regulation on the cell surface (Figure 6A).

Mammalian cells internalize plasma membrane-associated molecules, and sort these molecules through sorting endosomes into recycling or degradation pathways. To test whether there is also a change of LRP6 levels in endosomes after Dkk1 and/or Wnt3A treatment, we carried out endosome isolation after the cells were treated for 24 h. Similar to the changes of cell surface LRP6 levels, we found that Dkk1 treatment also induces LRP6 accumulation and blocks Wnt3A-induced LRP6 down-regulation in endosomes (Figure 6B), suggesting that Dkk1 treatment induces LRP6 accumulation; not only on the cell surface, but also in endosomes.

### Dkk1 stabilizes endogenous LRP6 and blocks Wnt3A-induced receptor down-regulation in breast cancer HCC1187

**Figure 7:**
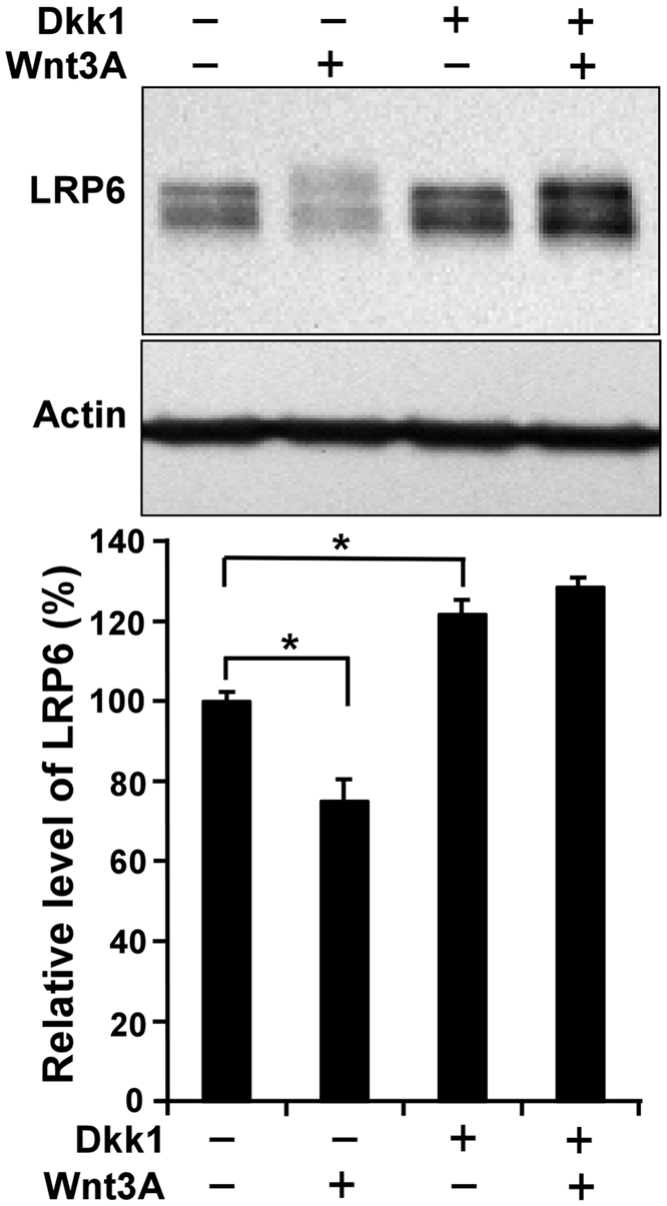
Dkk1 stabilizes endogenous LRP6 and blocks Wnt3A-induced LRP6 down-regulation in breast cancer HCC1187 cells. HCC1187 cells were incubated with 25% of L cell control CM or 25% of Wnt3A CM in the presence or absence of 500 ng/ml of recombinant Dkk1 protein for 24 h. The level of LRP6 was then examined by Western blotting. Samples were also probed with the anti-actin antibody to verify equal loading. The pixels for each band were measured, normalized and plotted. Data are mean values of three independent experiments with the SE values indicated by error bars. * P<0.05 versus control.

To confirm the role of Dkk1 in LRP6 protein regulation, we studied the effects of Dkk1 on the stability of endogenous LRP6. We found that, compared to several other breast cancer cell lines, HCC1187 cells express a relatively high level of endogenous LRP6 (data not shown). As expected, treatment of HCC1187 cells with recombinant Dkk1 protein for 24 h resulted in the accumulation of endogenous LRP6 and blockage of Wnt3A-induced receptor down-regulation (Figure 7).

### LRP6 degradation requires caveolae-mediated endocytosis

**Figure 8:**
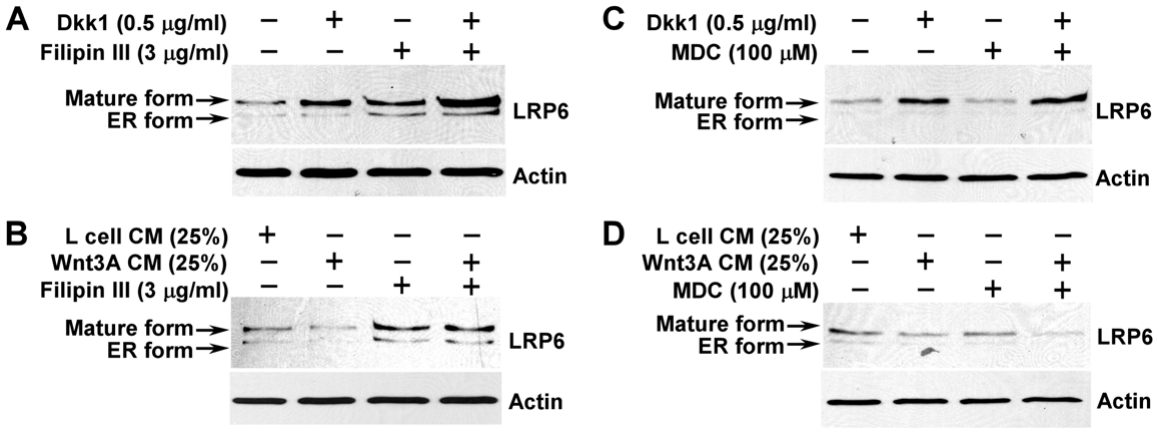
Effects of Filipin III and monodansylcadaverine on LRP6 turnover. HT1080 cells stably transduced with HA-tagged LRP6 were incubated with 0.5 µg/ml of recombinant Dkk1 protein (A, C) or 25% of Wnt3A CM (B, D), and treated with Filipin III (3 µg/ml) (A, B) or monodansylcadaverine (MDC) (100 µM) for 24 h. The level of LRP6 was then examined by Western blotting with the anti-HA antibody. Samples were also probed with the anti-actin antibody to verify equal loading.

Recent studies have demonstrated that the endocytic trafficking of Wnt proteins and their receptors is required for activation of the canonical Wnt signaling pathway [22]–[29]. To test a potential role for endocytosis in LRP6 turnover, we studied the effects of pharmacological inhibitors of clathrin-mediated and caveolae-mediated endocytosis on LRP6 levels. We found that treatment with Filipin III, an inhibitor of caveolae-mediated endocytosis, induced LRP6 accumulation, enhanced Dkk1-induced LRP6 stabilization, and blocked Wnt3A-induced LRP6 down-regulation in HT1080 LRP6 cells (Figure 8A & 8B). We also found that the clathrin-dependent endocytosis inhibitor monodansylcadaverine (MDC) had no effect on LRP6 levels (Fig. 8C & 8D). Together, these results indicate that LRP6 turnover is dependent on caveolae-mediated endocytosis, but not clathrin-mediated endocytosis.

## Discussion

Dkk1 is a major secreted Wnt signaling antagonist. Dkk1 binds to LRP6 and prevents LRP6-Wnt-Fz complex formation in response to Wnts. When it interacts with Kremen1/2, Dkk1 promotes LRP6 degradation [11]–[13]. In the present study, we have identified a novel role for Dkk1 in the absence of Kremen proteins; stabilizing LRP6 while inhibiting Wnt/LRP6 signaling. Importantly, we also demonstrated that there is a Wnt3A-induced LRP6 down-regulation, an effect that is blocked by Dkk1. Furthermore, we found that LRP6 degradation requires caveolae-mediated endocytosis.

Classically, ligand-induced cell surface receptor down-regulation has been recognized as a longer-term mechanism for termination of a signaling pathway [14], [15]. Down-regulation of growth factor receptors is essential for cells to control the extent of cell signaling, and defects in this process are often associated with enhanced cell proliferation and tumorigenesis [14], [15]. Wnts bind to their cell surface receptors Fz and LRP6 to activate the canonical Wnt signaling pathway. Recent studies demonstrated that the endocytic trafficking of Wnt/Wg proteins and their receptors is critical for the activation of the Wnt/Wg signaling pathway [22]–[29]. It has been reported that when cells are stimulated with Wnt3A CM, LRP6 is internalized, and the receptor is observed in the intracellular vesicles during a 10 min to 2 h time frame [29]. In the present studies, we demonstrate that treatment with Wnt3A CM induces down-regulation of stably transduced LRP6, which occurs in parallel with Wnt/LRP6 signaling decay. Our data suggest that there is a Wnt-induced LRP6 turnover after Wnt ligand stimulation. Furthermore, Khan *et al.* reported that there is a negative feedback regulation of LRP6 by which Wnt, through the downstream effector β-catenin, inhibits endogenous LRP6 expression at the transcriptional level [30]. Therefore, after activation of Wnt/LRP6 signaling, Wnt proteins could induce LRP6 down-regulation by enhancing LRP6 turnover and inhibiting new receptor synthesis.

Distinct from several families of secreted Wnt antagonists that bind Wnts, Dkk1 does not bind Wnt, but is a high affinity ligand for LRP6. However, the exact mechanism underlying the regulation of Wnt/LRP6 signaling by Dkk1 remains to be elucidated. It was reported that Dkk1 inhibits Wnt/LRP6 signaling by preventing Fz-LRP6 complex formation in response to Wnt [8]. Semenov *et al.* [31] demonstrated that Dkk1 inhibition of Wnt signaling is independent of LRP6 degradation, while Yamamoto *et al.* [32] reported that Wnt3A and Dkk1 regulate distinct internalization pathways of LRP6 to fine-tune Wnt signaling, and that clathrin-dependent internalization of LRP6 is required for the ability of Dkk1 to inhibit Wnt/LRP6 signaling. Very recently, Sakane *et al.* reported that Dkk1 induces the internalization of LRP6 to suppress LRP6 phosphorylation in the lipid raft and allows subsequent recycling of LRP6 so that it can be reused for signaling [33]. In our current studies, we found that Dkk1 is able to stabilize LRP6 while antagonizing Wnt/LRP6 signaling and blocks Wnt3A-induced LRP6 down-regulation. Thus, it is possible that while Wnt binding induces LRP6-Wnt-Fz complex formation and Wnt signaling activation, the LRP6 degradation and Wnt/LRP6 signaling decay take place after the internalization of LRP6-Wnt-Fz complexes. By preventing Wnt binding to LRP6, Dkk1 alone (without Kremen) can prevent LRP6-Wnt-Fz complex formation and internalization and stabilize LRP6 in a non-functional state (i.e. LRP6 is unable to induce Wnt signaling). Furthermore, Dkk1 alone binds to LRP6 and initiates LRP6 internalization [32], [33], which was confirmed in this study, showing that Dkk1 treatment increases LRP6 levels in endosomes.

Kremens interact with Dkk1 to promote LRP6 degradation [11]–[13]. Recently, it has also been reported that in the absence of Dkk1, Kremens can bind to LRP6, promote its cell surface localization, and stimulate LRP6 signaling [34], indicating that the absence or presence of Dkk1 determines whether Kremens will be activators or inhibitors of LRP6 signaling, respectively. In *Xenopus* axis duplication assays, it has been reported that Kremen2 synergizes with Dkk1 in inhibiting Wnt/LRP6 signaling [11]. However, studies also demonstrated that Kremen proteins are not universally required for Dkk1 function [35]. In the present study, we demonstrated that co-transfection of Kremen2 with Dkk1 can overcome the effects of Dkk1 alone on LRP6 stabilization, suggesting that the effect of Kremen2 on LRP6 stabilization and Wnt/LRP6 signaling is dominant over that of Dkk1. Furthermore, we found that co-transfection of Kremen 2 alone (without Dkk1) is sufficient to abolish LRP6 expression and Wnt/LRP6 signaling in HEK293 cells. Very recently, Schulze et al. studied Wnt signaling in the MC3T3-E1 osteoblast cell line, in which the endogenous expression of Kremen 2 and Dkk1 was undetectable [36]. They found that Dkk1 and Kremen 2 antagonize the activation of Wnt signaling, only when Wnt1 or Wnt3 expression plasmids are co-transfected. However, the activation of Wnt signaling is increased by Kremen 2, when a Wnt2 expression plasmid is used instead [36]. Therefore, future studies will be necessary to dissect the exact roles of Kremens in Wnt/LRP6 signaling.

Several recent studies have demonstrated the importance of endocytic trafficking of Wnt/Wg proteins and their receptors for the activation of the Wnt/Wg signaling pathway [22]–[29]. Using HEK293 cells and human cervical carcinoma HeLaS3 cells, Yamamoto et al. [29] reported that LRP6 is internalized via the caveolae-mediated endocytic pathway, which is required not only for Wnt-3a-induced internalization of LRP6, but also for the accumulation of β-catenin. Furthermore, they reported that clathrin-dependent internalization of LRP6 is required for the ability of Dkk1 to inhibit Wnt/LRP6 signaling [32]. On the other hand, using mouse fibroblasts (L cells), Blitzer and Nusse [25] reported that Wnt proteins are rapidly endocytosed by a clathrin- and dynamin-mediated process, and that interfering with clathrin-mediated endocytosis blocks Wnt signaling. Thus, Wnt may initiate LRP6 internalization and activate the Wnt/LRP6 signaling pathway through different endocytic pathways in different cell types. Here we found that LRP6 turnover requires caveolae-mediated endocytosis in HT1080 fibrosarcoma cells. Further studies will be required to define the precise mechanisms underlying Wnt-induced LRP6 trafficking and signaling.

**Figure 9:**
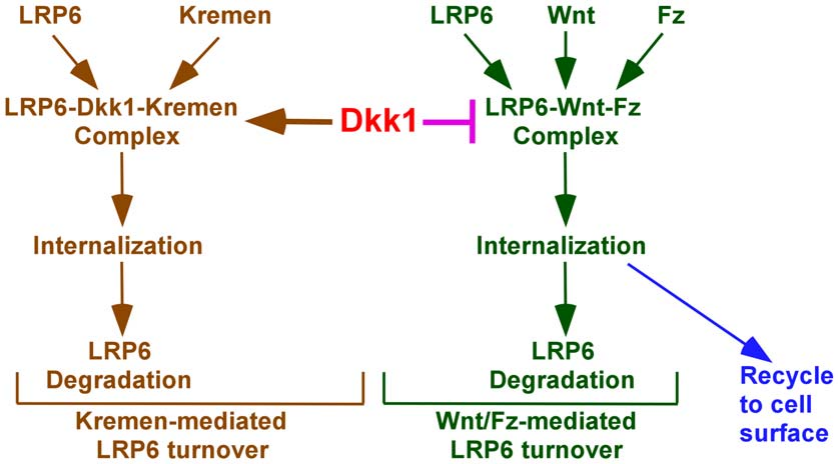
Model depicting the LRP6 turnover pathways. The first pathway is mediated by Kremen. In the presence of Kremen, LRP6 antagonist Dkk1 serves as a bridge between LRP6 and Kremen and induces LRP6 internalization and degradation, terminating Wnt signaling. Furthermore, Kremen might directly bind to LRP6 and induce LRP6 degradation. The second pathway is mediated by Wnt/Frizzled. While Wnt binding to its receptors LRP6 and Frizzled (Fz) induces LRP6-Wnt-Fz complex formation, the endocytosis and Wnt/LRP6 signaling decay of this complex occur after Wnt signaling activation. Dkk1 blocks Wnt binding to LRP6 and then inhibits LRP6 turnover mediated by Wnt/Fz.

In summary, based on our current report and other studies [11]–[13], [25], [29]–[31], [33], we propose that there are two LRP6 turnover pathways: Kremen-mediated LRP6 degradation, and Wnt/Fz-mediated LRP6 down-regulation (Figure 9). The former removes LRP6 from the cell surface and makes LRP6 unavailable for Wnt signaling, and the latter desensitizes Wnt signaling after activation of Wnt signaling.

## Materials and Methods

### Materials

Plasmid pCS-mKremen2 containing the full-length mouse Kremen2 cDNA and plasmid pCS-Myc-hLRP6 containing the full-length human LRP6 cDNA were provided by Dr. Christof Niehrs (Deutsches Krebsforschungszentrum, Heidelberg, Germany). The construction of the pcDNA3-Myc-Dkk1 plasmid containing the full-length human Dkk1 cDNA has been described before [37]. Human recombinant Dkk1 and sFRP1 proteins were obtained from R&D Systems. The preparation of mouse recombinant Mesd protein has been described before [37]. The monoclonal anti-LRP6 antibody was purchased from Santa Cruz. The monoclonal anti-β-catenin antibody was purchased from Transduction Laboratories. The monoclonal anti-c-Myc antibody was from Roche. The monoclonal anti-Rab5 antibody was purchased from BD Transduction Laboratories. The monoclonal anti-HA antibody was generated as previously described [38]. Peroxidase labeled anti-mouse antibody and ECL system were purchased from Amersham Life Science. All tissue culture media, serum, and plastic-ware were obtained from Life Technologies, Inc. Immobilon-P transfer membrane was purchased from Millipore. Rainbow molecular weight markers were purchased from GE Healthcare. Proteinase inhibitor cocktail Complete™ was obtained from Boehringer Mannheim. Filipin III and monodansylcadaverine (MDC) were purchased from Sigma.

### Cell culture and transfection

LRP6-transduced HT1080 cells have been described before [21]. HCC1187 cells, HEK293 cells, Wnt3A-secreting L cells, Wnt5A-secreting L cells, and control L cells were obtained from American Type Culture Collection. LRP6-transduced HT1080 cells, Wnt3A-secreting L cells and Wnt5A-secreting L cells were cultured in Dulbecco's minimum essential medium containing 10% fetal bovine serum, 2 mM L-glutamine, 100 units/ml penicillin, 100 µg/ml streptomycin and 350 µg/ml G418, and maintained at 37°C in humidified air containing 5% CO_2_. MDA-MB-231 cells, HEK293 cells and L cells were cultured in Dulbecco's minimum essential medium supplemented with 10% fetal bovine serum, 2 mM L-glutamine, 100 units/ml penicillin, 100 µg/ml streptomycin. For transfection, HEK293 cells were trypsinized and seeded into 6-well plates (2.0×10^5^ cells/well) without antibiotics. After 24 h, cells were transfected with various plasmids using FuGENE 6 (Roche) according to the manufacturer's specifications. Cells were harvested for analysis 48 h after transfection.

### Wnt3A, Wnt5A and Dkk1-conditioned media

Wnt3A-conditioned medium (CM), Wnt5A CM and L cell control CM were prepared according to the manufacturer's specifications. For Dkk1 CM, HEK293 cells in 10 cm dishes were transfected with 4 µg of pcDNA3-Myc-Dkk1 or the control pcDNA3 vector using FuGENE 6 according to the manufacturer's specifications. The media were changed with fresh Dulbecco's minimum essential medium containing 10% fetal bovine serum 24 h after transfection. After further 24 h incubation, the media were collected, centrifuged to remove cell debris, and stored at −80°C.

### Luciferase reporter assay

HEK293 cells were plated into 6-well plates. For each well, 0.1 µg of the TOP-FLASH TCF luciferase construct (Upstate Biotechnology) was co-transfected with LRP6-, Dkk1-, Kremen2-expressing vector, or empty pcDNA3 vector. A β-galactosidase-expressing vector (Promega, Madison, WI) was included as an internal control for transfection efficiency. After 48 h, cells were lysed and both luciferase and β-galactosidase activities were determined with enzyme assay kits (Promega). Luciferase activity was normalized to the activity of the β-galactosidase.

### Western blotting

HEK293 cells transiently transfected with Myc-tagged LRP6 or HT1080 cells stably transduced with HA-tagged LRP6 in 6-well plates were lysed in 0.5 ml of lysis buffer (phosphate-buffered saline containing 1% Triton X-100 and 1 mM PMSF) at 4°C for 30 min. Equal quantities of protein were subjected to SDS-PAGE under non-reducing conditions. Following transfer to immobilon-P transfer membrane, successive incubations with anti-Myc antibody, anti-HA antibody, anti-LRP6, or anti-β-catenin, and horseradish peroxidase-conjugated secondary antibody were carried out for 60 min at room temperature. The immunoreactive proteins were then detected using the ECL system. Films showing immunoreactive bands were scanned by Kodak Digital Science DC120 Zoom Digital Camera.

### GST-E-cadherin binding assay

Plasmid pGST-E-cadherin was provided by Dr. Gail Johnson (University of Alabama at Birmingham, Alabama). The GST-E-cadherin binding assay was carried out exactly as previously described [39], [40]. Uncomplexed β-catenin present in 200 µg of total cell lysate was subjected to SDS-PAGE and detected using a monoclonal antibody to β-catenin.

### Flow cytometric analysis of cell surface LRP6

Cell surface LRP6 measurement was carried out using the method described previously [21]. Briefly, HT1080 cells stably transduced with HA-tagged LRP6 were seeded at 1.0×10^6^ cells per T25 flask and cultured overnight before experiments. Cells were washed and incubated with or without human Dkk1 recombinant protein (1 µg/ml) for 24 h. Cells were then detached by incubation with nonenzymatic cell dissociation solution (Sigma). Successive incubations with affinity-purified anti-HA IgG (25 µg/ml) and goat anti-mouse Ig-FITC were carried out at 4°C for 45 min each. Background fluorescence intensity was assessed in the absence of primary antibody and subtracted from all samples. Mean fluorescence values were obtained from at least triplicate analyses on a FACScalibur (BD Biosciences-PharMingen), and data were analyzed with Cell Quest software.

### Isolation of endosomes

To isolate endosomes, cells were grown to confluency (70 to 80%) in 10 cm dishes and treated as described previously for 24 h. The adherent cells were harvested in cavitation buffer (5 mM HEPES, pH 7.4, 3 mM CaCl2, 1 mM EGTA, and 250 mM sucrose) and homogenized (200 p.s.i. of nitrogen gas, for 5 min) in a cell disruption bomb (Parr Instrument Co., Moline, IL, USA). The cell homogenate was centrifuged at 750×g for 5 min to remove unbroken cells and nuclei. The post-nuclear supernatant was loaded onto 1.045 g/ml Percoll (diluted in 250 mM sucrose) and centrifuged at 20,000×g (14,800 rpm) for 40 min using a Beckman Model Ti 70.1 rotor. A thick band between the supernatant and Percoll fraction was collected for each sample, diluted with dilution buffer (5 mM HEPES, pH 7.4, 3 mM CaCl_2_, 1 mM EGTA), and centrifuged at 14,000 rpm for 30 min at 4°C. The pellets were washed three times with cavitation buffer and centrifuged for 5 min between washes. The pellets were then re-suspended in the appropriate amount of cell lysis buffer (1X PBS+1% Triton-X 100) and incubated on ice for 30 min. Following the incubation, the homogenates were centrifuged at 14,000 rpm for 10 min at 4°C and the supernatants were transferred to 1.5 ml Eppendorf tubes.
